# Glycosaminoglycan-Protein Interactions by Nuclear Magnetic Resonance (NMR) Spectroscopy

**DOI:** 10.3390/molecules23092314

**Published:** 2018-09-11

**Authors:** Vitor H. Pomin, Xu Wang

**Affiliations:** 1 Department of BioMolecular Sciences, Division of Pharmacognosy and Research Institute of Pharmaceutical Sciences, School of Pharmacy, University of Mississippi, Oxford, MS 38677-1848, USA; 2 School of Molecular Sciences, Arizona State University, Tempe, AZ 85287-0112, USA

**Keywords:** glycosaminoglycan, GAG binding site, GAG-protein interactions, isotopic labeling, NMR, paramagnetic labeling

## Abstract

Nuclear magnetic resonance (NMR) spectroscopy is one of the most utilized and informative analytical techniques for investigating glycosaminoglycan (GAG)-protein complexes. NMR methods that are commonly applied to GAG-protein systems include chemical shift perturbation, saturation transfer difference, and transferred nuclear Overhauser effect. Although these NMR methods have revealed valuable insight into the protein-GAG complexes, elucidating high-resolution structural and dynamic information of these often transient interactions remains challenging. In addition, preparation of structurally homogeneous and isotopically enriched GAG ligands for structural investigations continues to be laborious. As a result, understanding of the structure-activity relationship of GAGs is still primitive. To overcome these deficiencies, several innovative NMR techniques have been developed lately. Here, we review some of the commonly used techniques along with more novel methods such as waterLOGSY and experiments to examine structure and dynamic of lysine and arginine side chains to identify GAG-binding sites. We will also present the latest technology that is used to produce isotopically enriched as well as paramagnetically tagged GAG ligands. Recent results that were obtained from solid-state NMR of amyloid’s interaction with GAG are also presented together with a brief discussion on computer assisted modeling of GAG-protein complexes using sparse experimental data.

## 1. Introduction

**Figure 1:**
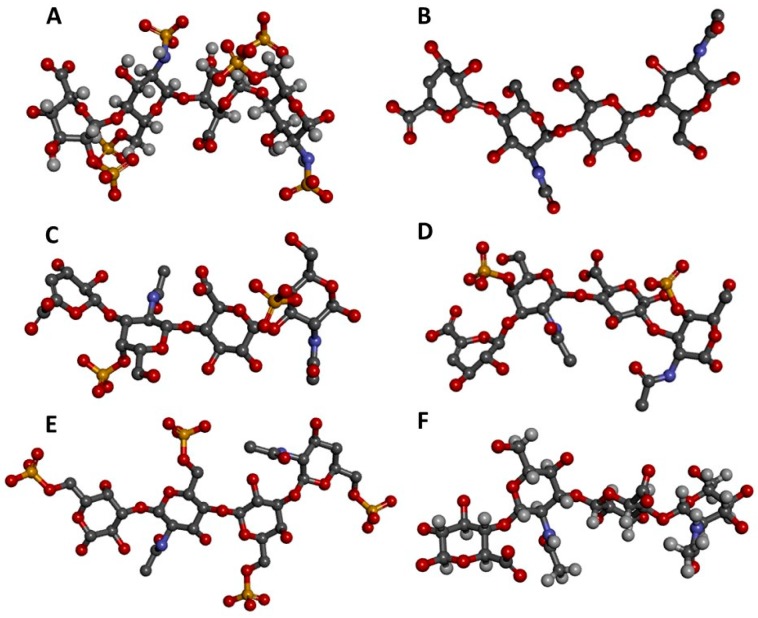
Three-dimensional representations of GAG tetrasaccharides taken from the protein data bank (PDB). (**A**) Heparin [IdoA2S-(α1-4)-GlcNS6S-(α1-4)-IdoA2S-(α1-4)-GlcNS6S] from 1HPN [7]; (**B**) Heparan sulfate [GlcA-(β1-4)-GlcNAc-(β1-4)-GlcA-(β1-4)-GlcNAc] from 3E7J [8]; (**C**) Chondroitin 4-sulfate (CS-A) [GlcA-(β1-3)-GalNAc4S-(β1-4)-GlcA-(β1-3)-GalNAc4S] from 1OFM [9]; (**D**) Dermatan sulfate (CS-B) [IdoA-(α1-3)-GalNAc4S-(β1-4)-IdoA-(α1-3)-GalNAc4S] from 1OFL [9]; (**E**) Keratan sulfate [Gal6S-(β1-4)-GlcNAc6S-(β1-3)-Gal6S-(β1-4)-GlcNAc6S] from 1KES [10]; and (**F**) Hyaluronan [GlcA-(β1-3)-GlcNAc-(β1-4)-GlcA-(β1-3)-GlcNAc] from 2BVK [11]. The atoms in the ball-stick representations are carbon (grey), nitrogen (blue), hydrogen (light grey); oxygen (red) and sulfur (yellow). Representations A and F are nuclear magnetic resonance (NMR) solution structures, and non-exchangeable protons are shown; the other representations are crystal structures so are shown without protons. Reprint with permission from MDPI *Pharmaceuticals* (*Basel*), *2018*, *11*(*1*) [12].

**Figure 2:**
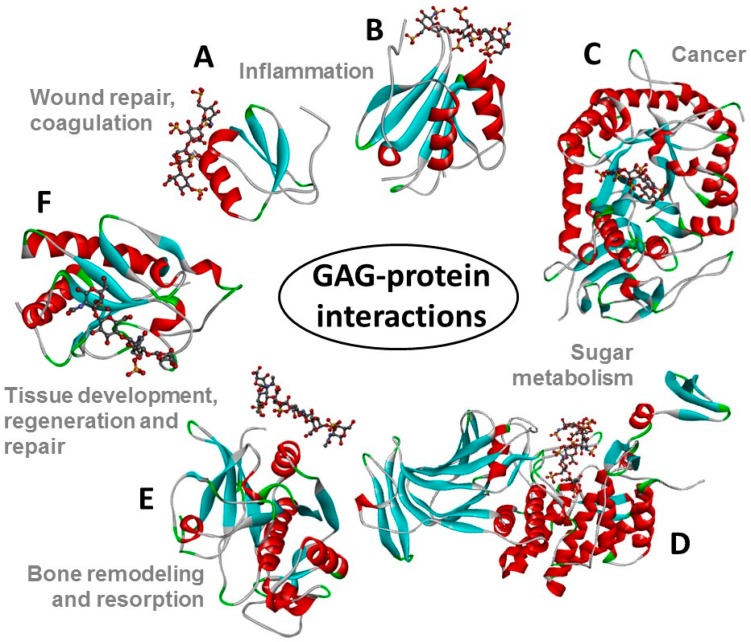
Structural representations from the crystal structures of some illustrative GAG-protein complexes in PDB: (**A**) macrophage inflammatory protein 1α (CCL3) monomer + heparin tetrasaccharide (from 5D65) [13], (**B**) Platelet factor 4 (CXCL4) dimer + fondaparinux (antithrombin-high affinity heparin pentasaccharide) (from 4R9W) [14], (**C**) human heparanase complex + heparin tetrasaccharide (from 5E9C) [15], (**D**) d-glucuronyl C5-epimerase + heparin hexasaccharide (from 4PXQ) [16], (**E**) Cathepsin K monomer + dermatan sulfate hexasaccharide (from 4N79) [17], and (**F**) Sonic Hedgehog monomer + CS-A tetrasaccharide (from 4C4M) [18]. The atoms of the GAG ligands represented in the ball-stick view are carbon (grey), nitrogen (blue), hydrogen (light grey); oxygen (red) and sulfur (yellow). In the proteins, the α-helices, β-sheets, loops and random coils are represented respectively in red, blue, green, and grey. The pathophysiological systems in which these complexes play a role are indicated by grey fonts in the panel. Reprint with permission from MDPI *Pharmaceuticals* (*Basel*), *2018*, *11*(*1*) [12].

Glycosaminoglycans (GAGs) are linear, anionic, high molecular weight (MW) polysaccharides that are composed of repeating disaccharide building blocks of alternating hexosamine and uronic acid/galactose units (Table 1, Figure 1). The monosaccharide composition and sulfation patterns of the disaccharide units of GAGs are used to classify GAGs into different categories (Table 1, Figure 1). GAGs have potent and varied biological activity. The formation of intermolecular complexes between GAGs and functional proteins is a requirement in most physiological and certain pathological events (Figure 2) [1,2,3,4,5,6]. These GAG-protein interactions and their consequential functions are prevalent in all tissues of the human body. 

Among all glycoconjugates in the glycocalix, GAGs are likely to be the most important player of the extracellular matrix in terms of protein interaction and regulation. This is because of two essential characteristics of these sulfated heteropolysaccharides: (i) they are ubiquitous to all eukaryotic cells; and, (ii) their highly complex structure gives rise to unique motifs that may have specific interactions with proteins. Hence, knowledge about the structural biology of GAG-protein interactions is extremely relevant. Three major goals exist in the current science of GAG-protein interactions: (i) identification of novel GAG sequences recognized specifically by proteins; (ii) identification of the potential GAG-binding site(s) in GAG-binding proteins; and, (iii) understand how GAG-induced changes in protein oligomerization and conformation regulate physiological activity.

One analytical technique that is capable of studying the various aspects of GAG-protein interactions including the three above-mentioned goals is nuclear magnetic resonance (NMR), whose versatility has made it a popular choice among researchers interested in GAG-binding proteins. NMR-oriented studies have been designed to elucidate multiple aspects of GAG-protein interactions, not only through the individual protein and ligand point-of-views but also through the perspective of the resultant complexes in solution, including their conformational fluctuations from the ns-to-ms timescale.

In this review, we want to offer an overview ofthe potentialsof this analytical technique for advancing studies of GAG-protein interactions. Although discussion on the most common NMR-based methods that are utilized to investigate GAG-protein interactions, such as chemical shift perturbation, saturation transferred difference, and transferred nuclear Overhauser effect, are presented, attention is also given to newer methods that have been developed lately. These up-to-date methods include water-ligand observed via gradient spectroscopy, isotopic enrichment and paramagnetic labeling of GAG ligands of well-defined chemical structures, characterization of lysine and arginines of GAG-binding sites, dynamics of the GAG-protein complexes, and solid-state NMR for resultant complexes with low solubility. Finally, we discuss how these NMR-based findings of GAG-protein interactions can be used to create molecular models of the complexes to better understand the structure-activity relationship of GAG-protein interactions.

## 2. Common NMR Experiments

### 2.1. Chemical Shift Perturbation 

Chemical shift perturbation (CSP) or chemical shift mapping is perhaps the mostly used NMR method in GAG-protein interaction studies [19]. It is generally carried out on ^15^N-labeled proteins and allows for GAG-binding sites in proteins to be identified. The protein atomic chemical shifts of interest are usually obtained through the ^15^N-edited heteronuclear single quantum coherence (HSQC) spectra of ^15^N-labeled proteins and medium-sized GAG oligosaccharides whose composition can be heterogeneous. To determine the GAG-binding site, multiple HSQC spectra of the protein with increasing concentrations of the ligand are recorded. These spectra allow for the ligand induced chemical shift changes in protein atoms to be quantified. In particular, the ^15^N-HSQC experiment measures the chemical shift changes of backbone amide proton and nitrogen (Δδ_H_ and Δδ_N_) in a residue specific fashion. The magnitudes of the changes are often diagnostic of ligand binding sites as larger changes are interpreted as a consequence of closeness of the atoms to the ligand. But, exceptions can exist. For example, when aromatic rings or charges approach the amide groups, this can sometimes exhibit a larger chemical shift change than expected just from the distances. Nonetheless, the magnitude of CSP for amide ^1^H and ^15^N resonances is usually quantified while using the empirical nonlinear Equation (1), where Δδ_HN_ and Δδ_N_ are the observed CSP for ^1^H and ^15^N, respectively.

Δδ = [(Δδ_HN_)^2^ + (Δδ_N_ × 0.12)^2^]^1/2^ (1)

The weighting factor 0.12 reflects the difference in chemical shift dispersion of ^1^H and ^15^N in folded proteins [20]. These data can be easily used to determine the dissociation constant (*K*_d_) of the intermolecular complex with a binding stoichiometry of one-to-one using Formula (2), where x is the total ligand concentration, Δδ is the observed chemical shift change, [Protein] is the total protein concentration, and Δδ_max_ is the maximum GAG-induced chemical shift change for the atom.

Δδ = (Δδ_max_/2[Protein])·{(*K*_d_ + x + [Protein]) − [(*K*_d_ + x + [Protein])^2^ − (4[Protein]x)]^1/2^} (2)

As discussed in Section 7, data derived from CSP can be used as input in computational docking for generating the molecular model of the resultant complex. 

**Figure 3:**
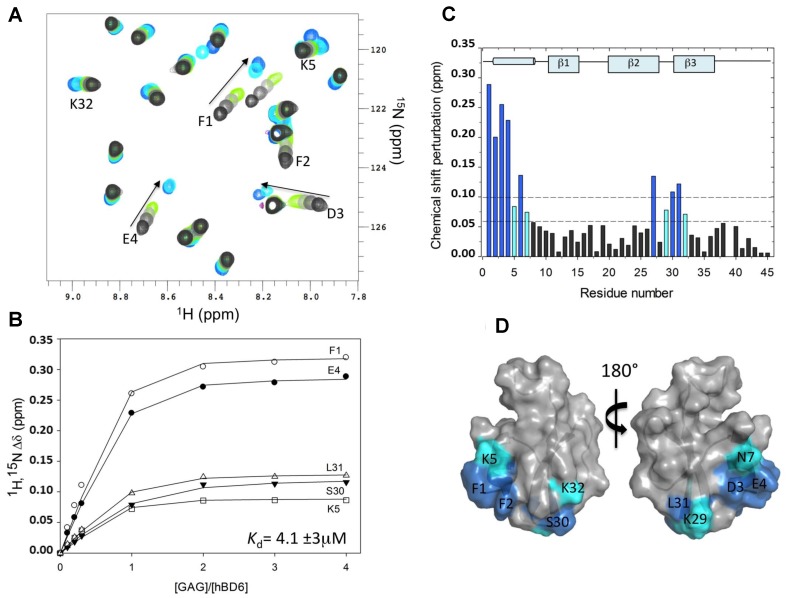
Interaction of human beta-defensin 6 (hBD6) with fondaparinux (Fx) by chemical shift perturbation (CSP) analysis. (**A**) A section of the ^15^N-edited heteronuclear single quantum coherence (^15^N-HSQC) spectra of the free hBD6 (black) and in the presence of heparin pentasaccharide Fx. The arrow indicates the direction of binding-induced chemical shift changes; (**B**) Representative binding profile for calculating the binding constant of hBD6-Fx interactions; (**C**) CSP map of hBD6-Fx interactions. The change seen for individual residues were judged in cutoffs (dotted lines) of 0.06 (light cyan) and 0.10 (dark cyan) ppm. The sequence-specific secondary structural elements are shown on the top of the CSP map; (**D**) Surface representation of hBD6 residues that are significantly perturbed on heparin pentasaccharide binding. The perturbed residues are shown in blue and cyan. Republished with permission of ASBMB *J. Biol. Chem.* from Unique properties of human β-defensin 6 (hBD6) and glycosaminoglycan complex: sandwich-like dimerization and competition with the chemokine receptor 2 (CCR2) binding site. de Paula V.S., Pomin V.H., Valente A.P.; 289, 33, 2014; permission conveyed through Copyright Clearance Center, Inc. [21].

As an example of the CSP application, the binding region of fondaparinux (Fx) on human beta-defensin 6 (hBD6) mapped by this method is here described [21]. Fx is a synthetic and highly sulfated heparin pentasaccharide that is commonly exploited as heparan sulfate mimetic in NMR-based GAG-protein interaction studies. HS is believed to be the major physiological target for most of the GAG-binding proteins, including the defensin hBD6. Isolation of well-defined oligosaccharides of heparan sulfate is difficult and since Fx is commercially available, this ligand becomes a preferable choice for research. Figure 3A shows a partial but detailed region of the ^15^N-HSQC NMR spectra of hBD6 recorded as a function of the Fx concentration: from a 0 (black) to 4:1 Fx:hBD6 ratio (cyan). NMR titration experiments revealed large changes in hBD6 chemical shifts in a fast exchange equilibrium between free and Fx-bound hBD6, as demonstrated by a gradual shift in peak positions seen by the different ^15^N-HSQC spectra recorded with increasing concentrations of heparin ligand. The quantitative analysis using the ^1^HN and ^15^N chemical shift changes for a subset of hBD6 resonances upon titration with Fx using the above-discussed formulas yielded a *K*_d_ value of 4.1 (±2.9) μM (Figure 3B). Figure 3C depicts the CSP map of Fx on hBD6 upon complete saturation (Fx:hBD6 molar ratio of 4:1) plotted as a function of the residue number of hBD6 for the Fx titration.

From the work depicted in Figure 3, the hBD6 residues most sensitive to Fx binding were determined. They are located in the α-helix (amino acids F1, F2, D3, E4, K5, C6, N7) and in part of the β2 and β3 strands (amino acids C27, Q28, K29, S30, L31, K32) of the defensin. Three lysines (K5, K29, and K32) out of seven have backbone ^15^N and ^1^H resonances significantly affected by the binding of Fx (Figure 3). These three perturbed lysine residues were reported to be part of the same heparin binding motif in hBD6 [21]. These basic amino acids perturbed in the CSP method indicate that electrostatic interactions are a major contributor to Fx binding to hBD6. Figure 3D shows the Fx binding site mapped onto the hBD6 structure (PDB ID: 2LWL). This dataset was revisited herein with permission from [21].

It is worth pointing out that CSP is not only reflective of direct protein-ligand interactions since CSP can be also influenced by other factors, such as changes in protein conformation due to binding and/or on the chemical environment of the mapped region of the protein. In addition to that, as commented above, when aromatic rings and/or charged groups approach amides, magnitude of the CSP may not be solely related to physical contact and this would increase complexity during data interpretation, leading to artifactual results. An alternative to enhance precision in the GAG-protein interaction study is to perform additional paramagnetic relaxation enhancement (PRE) experiments to complement the CSP methodology. The PRE method is described below at Section 3.3. Experiments designed to look at dynamics and orientations of side chains of lysines and arginines should also be helpful in determining the GAG-binding residues.

### 2.2. Saturation Transfer Difference

**Figure 4:**
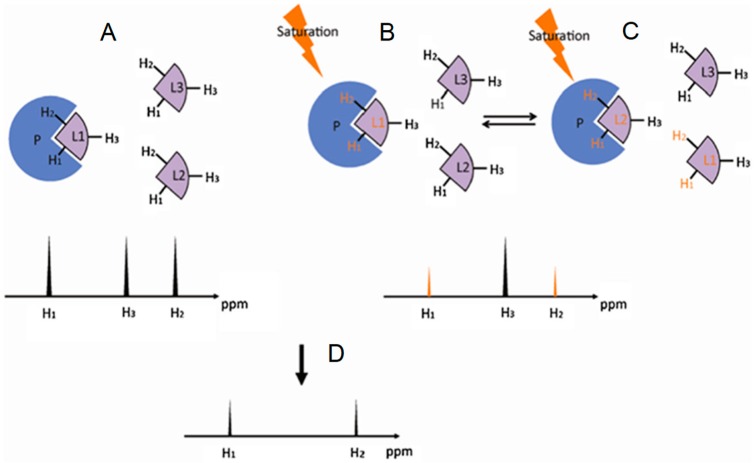
Schematic representation of the saturation transfer difference (STD) experiment. (**A**) The atoms of the ligand free in solution, in this case H_1_, H_2_ and H_3_, generate NMR signals whose intensities are proportional to their abundance; (**B**) Upon interaction, the saturated energy-excitement (orange arrow) on the protein will be transferred to the bound atoms (orange fonts); (**C**) The on-off behavior of the protein-ligand complex in solution leads to free but magnetized ligands. The intensities of the NMR signals will reflect the difference between the non-saturated (black peak) and the saturated atoms (orange peaks); The resultant STD spectrum (**D**) will be generated by subtracting the saturated spectrum (**B**,**C**) from the reference spectrum (**A**). Modified with permission from Springer *Anal. Bioanal. Chem.* License in conditions provided by Springer Nature and Copyright Clearance Center, Inc. [25].

Saturation transfer difference (STD) is a NMR experiment designed to map ligand protons that are involved in the interaction with protein [22,23,24,25]. In this method a group of protein protons, whose resonance frequencies do not overlap with any resonance frequency of the ligand, is selectively saturated. The extensive dipole-dipole interaction network in a protein allows the saturation energy to be spread to other protein protons as well as bound ligands, resulting in decreases in the signal intensities of the ligand protons in contact with the protein. Figure 4 illustrates the principle behind this NMR method. Because ligand proton signals are far easier to detect and STD is usually carried out under excess ligand conditions, STD is most frequently used to investigate the GAG-protein complexes through the GAG (ligand) perspective.

**Figure 5:**
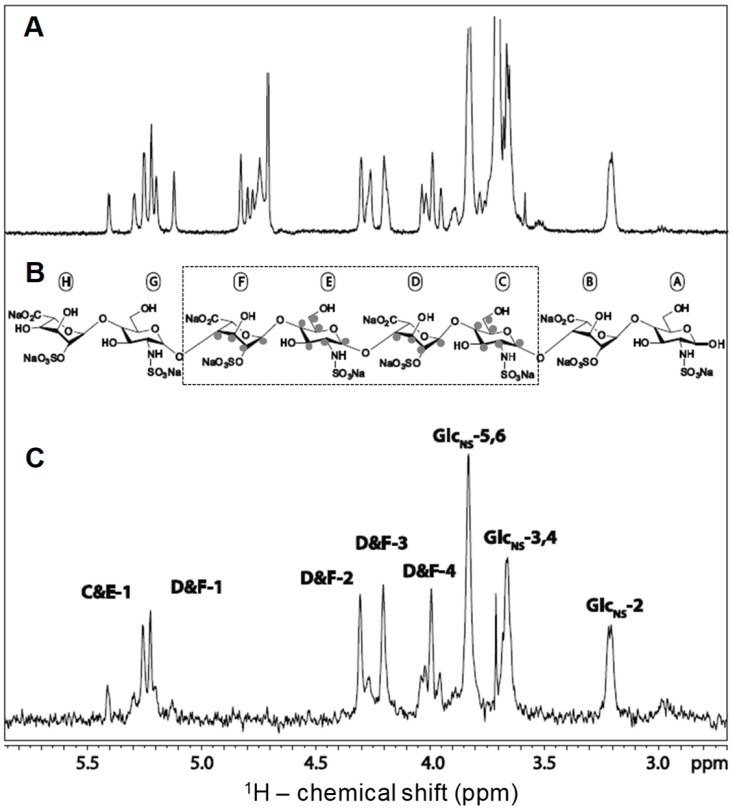
NMR assignments of the STD spectrum obtained from the complex of a heparin octasaccharide and fibroblast growth factor 2 (FGF2). (**A**) Reference spectrum recorded in the presence of FGF2, but without saturating the protein with magnetization; (**B**) Structure of the heparin octasaccharide utilized in the analysis. This ligand is composed of alternating IdoA2S and GlcNS units. See Table 1 for abbreviations. The internal tetrasaccharide highlighted by the dashed box indicates the binding motif recognized by the STD experiment; and, (**C**) The grey dots on the structural units indicate the signals assigned in the STD spectrum. Adapted with permission from Springer *Anal. Bioanal. Chem.* License in conditions provided by Springer Nature and Copyright Clearance Center, Inc. [25].

STD has been used to investigate the GAG specificity of numerous GAG-binding proteins, including basic fibroblast growth factor (bFGF or FGF2). Crystal structure of FGF2 with heparin showed IdoA2S-GlcNS disaccharide units are essential for strong FGF2-GAG interactions and this was later confirmed by Yu et al. through STD (Figure 5) [25]. In Figure 5A, the 1D ^1^H reference spectrum of 30 μM of FGF2 and 370 μM of a synthetic heparin octasaccharide composed of only IdoA2S-GlcNS disaccharide units (Figure 5B) is shown. The STD spectrum of the same sample is displayed in Figure 5C. Intense and narrow peaks can be noted in this STD spectrum, indicating significant interactions between FGF2 and the ligand. The assignments of the ligand residues that are involved in the interaction revealed FGF2 has the strongest interaction with the internal region (dashed box) of the ligand. In particular, atoms on the 2-*O*- and *N*-sulfation side of the internal tetrasaccharide made of the units labeled as C, D, E, and F produced the strongest STD signals. 

### 2.3. Transferred Nuclear Overhauser Effect

**Figure 6:**
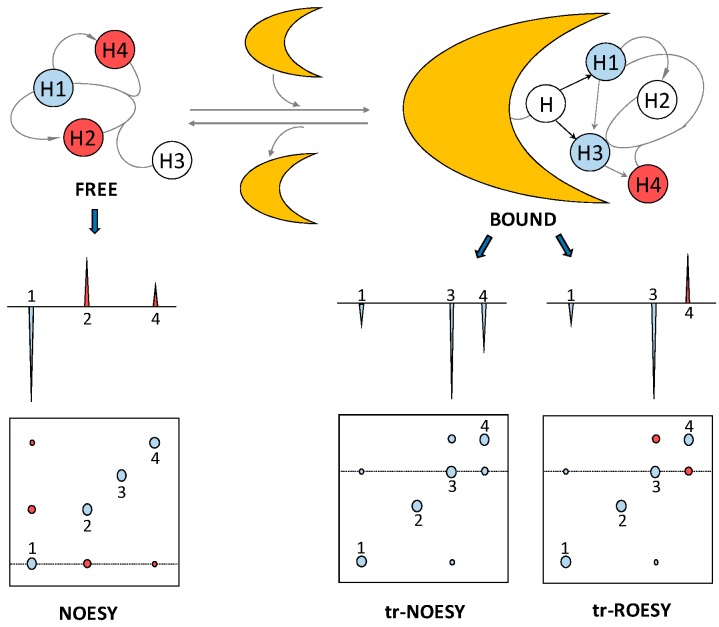
Schematic representation of the trNOESY and trROESY methods for an exchanging ligand-protein (yellow) complex. The NOE cross-peaks of a free ligand are positive (red) and display opposite sign to the negative (blue) diagonal (auto) peaks, but upon interaction with the protein in a trNOE experiment, these peaks also become negative due to the increase of effective rotational correlation time. The analysis of the negative cross-peaks provides information on the bound conformation of the ligand. The possible spin-diffusion-related trNOE cross-peaks can be identified by trROE where the alternation of the sign is diagnostic of the real and artifact peaks. Adapted with permission of RSC *MedChemComm* and Elsevier *Curr Opin Struct Biol* from NMR and molecular recognition. The application of ligand-based NMR methods to monitor molecular interactions. Unione L., Galante S., Diaz D., Cañada J., Jiménez-Barbero J. 5, 2014, and New structural insights into carbohydrate-protein interactions from NMR spectroscopy. Kogelberg H., Solís D., Jiménez-Barbero J. 13, 5, 2013; permission conveyed through Copyright Clearance Center, Inc. [27,28].

The bound-state conformation of a ligand, such as a GAG oligosaccharide, can be deduced through a method called exchange-transferred nuclear Overhauser effect (trNOE) [26]. Through the use of this NMR method, protein-induced changes in the intramolecular NOE signals of the ligand can be identified. This provides valuable information about the conformational changes of the GAG oligosaccharide upon interaction with the protein. Similar to STD, trNOE takes advantage of the changes in the rotational motion of the ligand upon binding to the larger macromolecule [27]. The rotational correlation time of small molecules such as medium- and short-sized GAG oligosaccharides lies on the ps timescale and the resultant NOE signals are small and positive. The correlation time of larger molecules, such as most of the GAG-binding protein and the resultant GAG-protein complexes, lies on the nanosecond time range and the associated NOEs are large and negative (Figure 6). Hence, the correlation time of a small ligand, GAG oligosaccharide in our case, changes considerably upon interaction with the protein partner. The consequential change of NOE cross-peak sign and magnitude can be used to elucidate the conformation of protein-bound ligands, even in the presence of excess unbound ligands.

Two of the biggest challenges for a trNOE experiment are the very narrow accessible kinetic window and the possible existence of spin-diffusion-associated effects. Regarding the first obstacle, for a trNOE experiment to be successful, the on-off rates must be fast relative to the spin relaxation rates. Otherwise, the NOE information acquired by the ligand at the protein interaction will be lost or decreased by relaxation properties before the ligand gets dissociated from the protein. This phenomenon is also dependent on affinity and thus the *K*_d_ of the complex. To optimize the conditions for the trNOE experiment, parameters such as temperature, ionic strength and protein:GAG oligosaccharide molar ratio must be carefully controlled. The second obstacle can be circumvented while using rotating-frame Overhauser effect (ROE). ROE cross-peaks have different signs depending on whether they are produced by direct or three-spin indirect effects [29] (Figure 6), allowing cross-peaks of direct interactions to be identified. Like the STD experiment, trNOE investigates the GAG-protein complexes through the GAG (ligand) perspective.

An example of the use of trNOE and trROE in a protein-GAG oligosaccharide investigation is the work of Künze et al., who studied the binding of a heparin tetrasaccharide to interleukin-10 (IL-10) [30]. The structure of the heparin fragment that was used in this work is ΔUA2S-GlcNS6S-IdoA2S-GlcNS6S (see Table 1 for abbreviations). ΔUA2S stands for 4,5-unsaturated 2-sulfated uronic acid. Both NOESY and ROESY spectra were collected at 150 ms mixing time on heparin tetrasaccharide alone and in the presence of IL-10. Experiments were recorded with 20-fold excess of the ligand. NOE/ROE signals of the GAG ligand at the free-state were close to zero and did not interfere with the signals observed in the bound state. In the absence of protein, positive NOEs were observed between protons H_2_ and H_3_, H_4_, and H_5_ of GlcNS6S at the non-reducing end disaccharide and between H_4_ and H_5_ of GlcNS6S of the reducing end. In addition, some small negative NOEs appeared between H_3_ and H_4_ of the IdoA2S, between H_3_ and H_4_ of GlcNS6S of the non-reducing end disaccharide and between H_6_ protons of both GlcNS6S. Multiple strong negative NOEs were observed in the heparin tetrasaccharide in the presence of 5 mol % of IL-10, aside from the single positive NOE between H_2_ and H_3_ of the GlcNS6S at the non-reducing end disaccharide. The set of trNOE observed in the experiment indicates proper molecular interaction between IL-10 and heparin since the average correlation time of the ligand has obviously increased significantly to produce negative NOE signals. trROE was also used to identify artifacts produced by spin-diffusion in the same study.

Another successful application of the trNOE technique is the recent publication of Gao et al. about the intermolecular interaction of Fx with the two N-terminal immunoglobulin domains (Ig1-2) of leukocyte common antigen-related protein (LAR) [31]. The temperatures and magnetic field was optimized so that the free ligand in solution produced no NOE cross-peaks (~30 °C at 800 MHz for Fx), therefore allowing for cross-peak intensities in trNOE to be interpreted while using the simple 1/r^6^ relationship. A set of NOE and trNOE-derived interproton distances (Table 2) were generated through these experiments and used for assessing conformational change of the ligand from the free- to the bound-state. Besides assessing information regarding the *^1^C_4_* chair and *^2^S_0_* skew-boat configurations of the IodA2S ring of Fx, the trNOE-derived data was also useful as conformational restraints in the docking process [31].

### 2.4. WaterLOGSY

**Figure 7:**
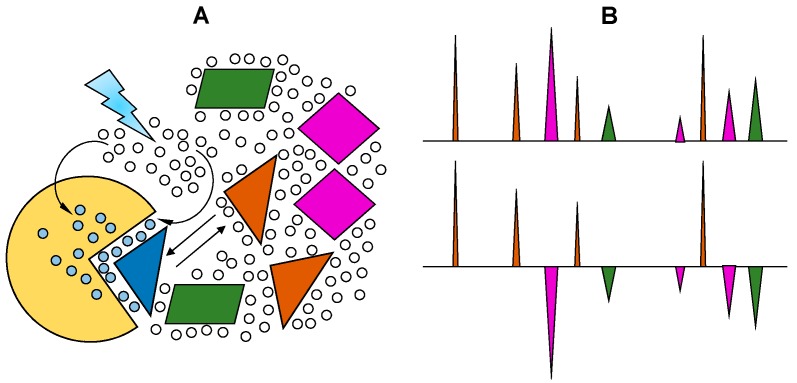
Schematic description of the waterLOGSY experiment. (**A**) The magnetization of the bulk water is selectively transferred, via the protein-ligand complex, to binding ligands (triangles). (**B**) Hypothetical representations of NMR spectra from a mixture (top) and from the resultant one-dimensional (1D) waterLOGSY spectrum (bottom), where binding and non-binding ligands can be straightforwardly identified. The positive-phase signals (brown peaks) are produced from binding ligands interacting with protein (blue and brown triangles) and cross-relax with protein-bound water, whose magnetization is selectively inverted. The peaks in negative-phase (green and purple peaks) come from the non-binding ligands which do not interact with inverted water molecules bound to the protein. Adapted with permission of RSC *MedChemComm* from NMR and molecular recognition. The application of ligand-based NMR methods to monitor molecular interactions. Unione L., Galante S., Diaz D., Cañada J., Jiménez-Barbero J. 5, 2014; permission conveyed through Copyright Clearance Center, Inc. [27].

Water-ligand observed via gradient spectroscopy (waterLOGSY) is an experiment whose mechanism is akin to STD and trNOE, but it is focused on identifying interactions between water and biomacromolecules [32]. The experiment was originally devised to confirm protein-ligand interactions mediated by water. It has since become a valuable tool in protein-glycan studies because interactions between proteins and glycans are often mediated by water, especially when the interactions are electrostatic in nature. Figure 7 illustrates the mechanism of waterLOGSY experiment. The experiment relies on the fact that trapping of water molecules in intermolecular niches between glycan and the target protein slows the motions of water molecules, leading to cross-relaxation with adjacent glycan and protein protons and the production of detectable cross-peaks in NOESY. In practice, the experiment is implemented as a one-dimensional selective NOE experiment where only the water magnetization is inverted. Although use of waterLOGSY in studies of protein-glycan interactions is common, an example involving GAG has been published only recently. Interestingly, in that study, Mandaliti et al. did not use the experiment to probe the ligand, but to identify protein residues that are involved in hyaluronan binding [33], providing a good demonstration of the versatility of the experiment. Although information from waterLOGSY is limited to hydration, it can often be used to create more accurate models and provide a deeper understanding of the energetics of binding. A quantitative utilization of the technique, referred to as LOGSY titration, was published recently. In this study the authors examined the waterLOGSY signal of the ligand at different protein concentrations to accurately identify ligand protons involved in water mediated interactions in the protein-ligand complex [34]. Such an experiment can be easily applied to the investigation of protein-GAG systems to pin point locations of water at the protein-GAG interface. 

## 3. Production of GAG Ligands for NMR

As mentioned above, native GAGs have two structural characteristics that make them not ideal for structural studies: (i) very complex structures with variable lengths (polydispersity); and, (ii) high MW chains that lead to multivalent binding and protein oligomerization and/or precipitation. As a result, only small oligosaccharides with defined structure can be used in high resolution structural studies. Currently, two methods are being employed to generate these defined GAG fragments: (i) purification of oligosaccharides derived from the enzymatic depolymerization of GAG from natural sources; and, (ii) synthesis of GAG-derived oligosaccharides through multiple chemical and enzymatic steps. Recently, we discussed the protocols that were used for chemoenzymatic synthesis and the GAG-like oligosaccharide libraries [35]. In the next section, we briefly discuss the three-step process used to prepare GAG oligosaccharides derived from natural sources.

Another obstacle for the NMR study of GAGs is that, because prokaryotic organisms that are capable of growing in minimal media do not produce sulfated GAGs, there is yet no simple method to produce isotopically enriched GAG polysaccharides. Despite these difficulties, some progress has been made to produce isotopically labeled GAG ligands for NMR study. The following sections are dedicated to explaining some of these methods as well as techniques for paramagnetically labeled GAG oligosaccharides for NMR studies.

### 3.1. Unlabeled Oligosaccharides

**Figure 8:**
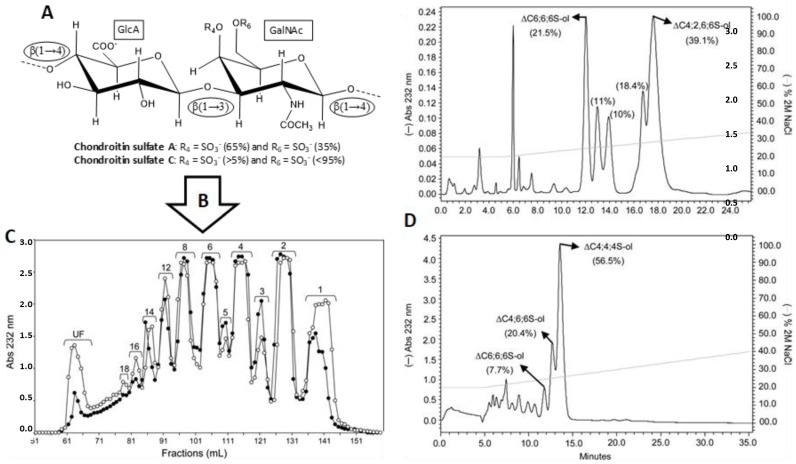
General scheme for production of GAG oligosaccharides of defined size and structures, exemplified by the various hexasaccharides of chondroitin sulfates A and C. (**A**) Structural representation of the chondroitin sulfate A and C. GlcA and GalNAc stand for glucuronic acid and *N*-acetylgalactosamine, respectively. Glycosidic linkages are depicted inside the ellipses. (**B**) After a 2-day digestion of chondroitin sulfate A derived from bovine trachea (btCS-A) or chondroitin sulfate C derived from shark cartilage (scCS-C) with a commercial preparation of chondroitinase C from *Flavobacterium heparinum*, the resultant mixture of fragments were applied in a SEC Bio-Gel P-10 column (**C**) for oligosaccharide fractionation (open circles for scCS-C and filled circles for btCS-A). Then, the pool of hexasaccharides was applied to a HPLC-SAX column for fractionation of isomers. (**D**) Both mixtures of hexasaccharides were obtained from peak 6 of their respective Bio-Gel P-10 chromatograms. The characterized structures of the fractionated isomers are the following: Top chromatogram: ΔC6;6;6S-ol (ΔUA-(β1-3)-GalNAc6S-(β1-4)-GlcA-(β1-3)-GalNAc6S-(β1-4)-GlcA(β1-3)-GalNAc6S-ol) and ΔC4;2,6;6S-ol (ΔUA-(β1-3)-GalNAc4S-(β1-4)-GlcA2S-(β1-3)-GalNAc6S-(β1-4)-GlcA-(β1-3)-GalNAc6S-ol) for scCS-C digestion. Bottom chromatogram: ΔC6;6;6S-ol, ΔC4;6;6S-ol (ΔUA-(β1-3)-GalNAc4S-(β1-4)-GlcA-(β1-3)-GalNAc6S-(β1-4)-GlcA(β1-3)-GalNAc6S-ol) and ΔC4;4;4S-ol (ΔUA-(β1-3)-GalNAc4S-(β1-4)-GlcA-(β1-3)-GalNAc4S-(β1-4)-GlcA(β1-3)-GalNAc4S-ol) for btCS-A digestion. The nomenclatures ΔUA, “S”, and -ol used for the structures stand, respectively, for Δ4,5 unsaturated uronic acid, sulfation group, which the number before “S” represent the ring position, and reduced sugars (open rings at the reducing-ends). The percentage of material yielded in each peak is given in parentheses. The NaCl gradient is shown with the continuous light grey line. Modified with permission from Oxford University Press. License in conditions provided by Oxford University Press and Copyright Clearance Center, Inc. [37].

Structurally homogeneous GAG fragments can be obtained by a general protocol involving three basic steps (Figure 8). First, the native GAG polymers (Figure 8A) are partially digested by GAG-degrading enzymes, such as hydrolases or lyases (Figure 8B). In this initial step, digestions must be very controlled in order to produce large amounts of medium-sized oligosaccharides, such as hexasaccharides and octasaccharides. Otherwise, the over-digested materials will produce mostly short-sized fragments, such as disaccharides and tetrasaccharides. Then, the mixture of digested materials is separated based on size while using size-exclusion chromatography (SEC) (Figure 8C). Lastly, the fragments of uniform sizes are re-fractionated based on structure and charge through strong-anion exchange (SAX) chromatography, which is usually performed in automated systems, such as high-pressure liquid chromatography (HPLC) (Figure 8D).

Since the resultant fragments are products of enzymatic digestions, sulfation patterns that are observed in the generated products are controlled by enzyme specificity and they may not reflect distribution of structural motifs in native GAG. This is a downside of the protocol because a particular structure of interest with important biological relevance may not be obtainable. Due to this limitation, GAG-protein investigations performed in the laboratory may not reflect the full spectrum of GAG motifs the protein is exposed to in vivo. Nonetheless, this method still remains the most practical way for generating structurally homogeneous ligands for further GAG-protein studies [36]. Another obstacle is the availability of GAG-digestive enzymes in reasonable amounts for studies. Although many GAG hydrolases and lyases are commercially available, their preparations are not consistent since they are isolates from natural sources. The inhomogeneous preparations of these enzymes impair reproducibility in structure and size distributions of the products. Although recombinant GAG-digestive enzymes exist and are more reliable in terms of homogeneity, they are not widely commercially available. 

### 3.2. Isotopically Labeled Oligosaccharides

Although there are great barriers in total chemical synthesis of GAGs, fortunately GAG precursors, such as heparosan [-4)-GlcNAc-(α1-4)-GlcA-(β1-]_n_, can be found in capsules surrounding *Escherichia coli* K5 and *Pasteurella multocida*. This has been exploited in biotechnology for chemoenzymatic synthesis of heparin- and heparin sulfate-like polymers [38]. Since the growth of *E. coli* K5 in minimal media supplemented with ^13^C-glucose and ^15^N-(NH_4_)_2_SO_4_ is easily achievable, ^13^C,^15^N-doubly labeled heparosan-derived heparan sulfate and heparin can be produced in small but sufficient quantities [39], and can be used as ligands in protein interaction studies [40]. No prokaryotic system has been reported so far to obtain chondroitin sulfate precursors, but efforts are in progress to obtain metabolically engineered *E. coli* for production of such precursors [41].

**Figure 9:**
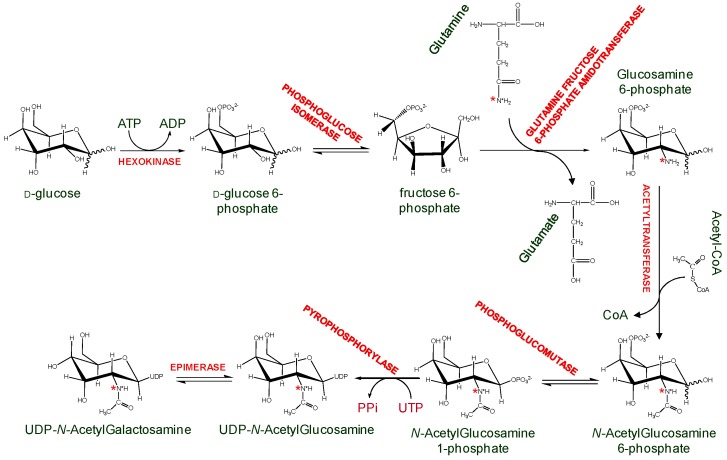
Schematic representation of the cytosolic biosynthesis of ^15^N-labeled UDP-*N*-acetylgalactosamine and UDP-*N*-acetylglucosamine, using side chain ^15^N-labeled glutamine as ^15^N donor. These nucleotide-*N*-acetyl-hexosamines will be further used inside the Golgi apparatus to synthesize GAG backbones in proteoglycans. The names of the enzymes are fully written in red while the substrates and products are mostly written in black. The ^15^N-labeling are indicated with the red asterisks throughout the reaction steps. Adapted with permission from Pomin et al. Analytical Chemistry 2010, 82, 4078–4088. Copyright 2018 American Chemical Society [42].

Another alternative is to generate isotopically labeled GAGs while using mammalian cells culture. An example uses commercially available side chain ^15^N-labeled glutamine to produce ^15^N-labeled amino sugars and consequently GAGs in Chinese Hamster Ovary cells and human lung endothelial cells. (Figure 9) [42]. These ^15^N-labeled GAGs were structurally characterized via ^15^N-NMR, whose signals have proven to be sensitive and diagnostic to a series of GAG structural features, such as lengths (disaccharides, oligosaccharides, and even high-order polymers), anomericity, glycosylation sites, hexosamine types, sulfation types (*O*-linked or *N*-linked) and positions, and neighboring uronate types (IdoA or GlcA) [43]. Although the GAG quantity produced in the above example was never sufficient for biochemical studies with GAG-binding proteins, attempts are underway to produce GAG in large scale cell cultures [44]. The ^15^N-labeling on these ligands can be exploited as a structural probe in GAG-protein interaction studies. In particular, ^15^N chemical shifts in these ligands were very sensitive to the surrounding environment, therefore protein-induced chemical shift perturbations on ^15^N resonances of these GAG ligands would offer a strategy to monitor interactions with GAG-binding proteins.

### 3.3. Paramagnetically Labeled Oligosaccharides

**Figure 10:**
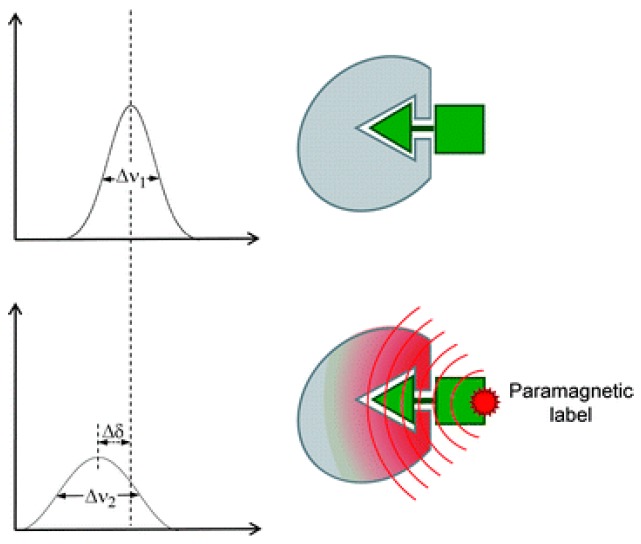
Paramagnetically tagged ligands affect the relaxation properties of their binding partners causing two major effects: pseudo-contact shifts (Δδ) and line-broadening (Δν_2_ > Δν_1_). These effects can be useful for mapping the binding site of the ligand. Adapted with permission of RSC *Nat. Prod. Rep.* from Application of NMR methods to the study of the interaction of natural products with biomolecular receptors. Calle L.P., Cañada F.J., Jiménez-Barbero J. 28, 6, 2011; permission conveyed through Copyright Clearance Center, Inc. [52].

Paramagnetically labeled GAG ligands have proven to be useful in structural studies of GAGs’ interactions with several different proteins [31,45,46,47,48,49]. The paramagnetic effect relies on the dipole-dipole interactions between unpaired electrons and surrounding atoms. Such interactions produce relaxation effects on nearby nuclei. The extent of the paramagnetism-induced increase in transverse relaxation of proton can be calculated using the following Equation (3):

(3) $$R_{2,\mathrm{pre}}=\frac{1}{15}{\left(\frac{\mu_{0}}{4\pi }\right)}^{2}\frac{\gamma_{H}^{2}g_{S}^{2}\mu_{B}^{2}S\left(S+1\right)}{r^{6}}\left(4\tau_{c}+\frac{3\tau_{c}}{1+\omega_{H}^{2}\tau_{c}^{2}}\right)$$

where R_2,pre_ is the increase in transverse relaxation rates due to the paramagnetic center, r is the distance between the paramagnetic center and the proton under analysis, γ_H_ is the gyromagnetic ratio of proton, g_s_ is the electron g factor for the unpaired electron, ω_H_ is the resonance frequency of the proton under analysis, and τ_c_ is the overall rotational correlation time of the whole system. This effect is called PRE and it can be accurately quantified using specific experiments [50,51]. However, paramagnetism can change more than the relaxation rate of a NMR signal. As seen in Figure 10, all paramagnetic labels can produce NMR signal line-broadening (Δν_2_ is bigger than Δν_1_) on nearby atoms, but paramagnetic center with asymmetric g-tensors and favorable relaxation properties can also produce chemical shift displacements (Δδ) in these atoms. These chemical shift changes are referred to as pseudo-contact shifts.

**Figure 11:**
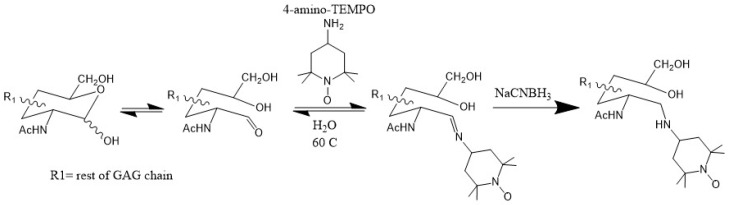
Reductive amination of the reducing end of glycans using 4-amino-TEMPO.

Since the magnitude of PRE is primarily dependent on the distance between the paramagnetic center and nearby atoms, data from this technique are simpler to interpret than CSP, which is also sensitive to allosteric effects. Therefore, PRE is a reliable validation of CSP data. Another key advantage of PRE is that the effective range of the paramagnetic effect can be up to 20 Å. This is ideal for detecting long-range or transient contacts between GAGs and proteins. In several works [45,46,47], GAG ligands have been functionalized with the paramagnetic tag TEMPO (2,2,6,6-tetramethylpiperidine-1-oxyl) through the reductive amination of the reducing end of the oligosaccharide (Figure 11). This method has the distinct advantage of being specific to the reducing end of the GAG ligand, allowing for the location of the reducing end of the ligand to be identified. 

**Figure 12:**
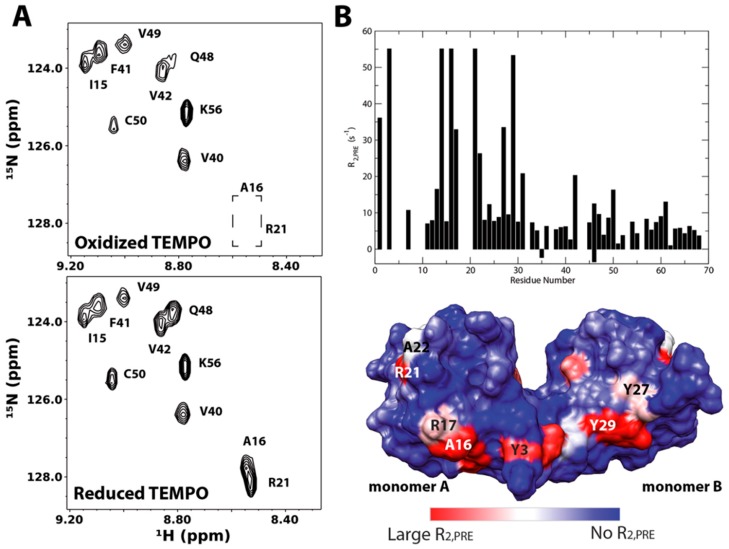
(**A**) ^15^N-HSQC spectra of RANTES in the presence of oxidized (top panel) and reduced (bottom panel) forms of TEMPO-labeled chondroitin sulfate hexasaccharide CS6;4;4. (**B**) Quantitative measurements of paramagnetic TEMPO-induced transverse relaxation (R_2,pre_). Graph and surface plot of amide proton R_2,pre_ produced by TEMPO-labeled CS6;4;4. Residues Y14, A16 and R21 disappeared upon addition of the ligand, but re-appeared upon its reduction. They are given the same magnitude of R_2,pre_ as residue Y3, which showed the largest PRE effect. Reproduced with permission of RSC New developments in NMR. NMR in Glycoscience and Glycotechnology. Chapter 11. Xu Wang, page 264, 2017 [36].

A particular interesting application of the technique was provided by Deshauer et al [45]. In this study, TEMPO-tagged chondroitin sulfate hexasaccharides were used to investigate GAG interactions of the chemokine CCL5/RANTES. Figure 12 revisits the data. The ^15^N HSQC spectra of RANTES in the presence of either oxidized or reduced TEMPO-labeled chondroitin sulfate hexasaccharide CS6;4;4 (GlcA-(β1-3)-GalNAc6S-(β1-4)-GlcA-(β1-3)-GalNAc4S-(β1-4)-GlcA(β1-3)-GalNAc4S-TEMPO) are shown in Figure 10A. When comparing the ^15^N-HSQC spectra, several signals have disappeared upon the oxidation of TEMPO. Those belonging to residues A16 and R21 are highlighted in the upper spectrum. This indicates these residues are in close contact with the paramagetically tagged ligand. Quantitative measurements of the ^1^H PRE of the amide protons were carried out (graph on Figure 12B) and used to identity of the binding site on RANTES (Figure 12B). This approach was also similarly employed in investigations of GAG binding by the bacterial adhesion decorin-binding proteins A and B [47,53].

## 4. Characterizing Lysine and Arginine Residues in GAG-Binding Sites

### 4.1. Direct Observation of Lysine Amine Signals

Because of their ability to participate in ionic interactions with GAGs, lysines and arginines are the most important residues in GAG-binding sites. However, studying these side chains is not routine. Specifically, the positively charged functional groups at the termini of their side chains contain labile protons that are not usually NMR detectable. Since interactions are mediated by these functional groups, their invisibility in NMR meant that much of GAG-protein interactions remains undetectable. This problem is especially acute with lysines since protons on their terminal amine are highly exchangeable, whereas some information on arginine can usually be gleaned from Hε-Nε of arginine while using only ^15^N-HSQC.

The group of Junji Iwahara has contributed greatly to NMR investigations of lysine side chain amine groups. In particular, Iwahara and co-workers have shown that the heteronuclear in-phase single quantum coherence experiment (HISQC) is more sensitive at detecting side chain amine signals because of its ability to reduce the relaxation effect of hydrogen exchange on ^15^N atoms [54]. The Iwahara group also developed much of the theory behind the relaxation of ^15^N atoms in -NH_3_^+^ groups and showed that DNA binding to a protein changes dynamics of some of its lysine and arginine residues [55,56]. Their methodology is easily adaptable to GAG-binding proteins. The first instance of these is reported by Sepuru et al. in the study of the interactions of chemokines CXCL1 and CXCL5 with heparin. They noted that, in the absence of GAG octasaccharides, very few lysine side chain amine signals from the two chemokines can be seen even at pH 5.7 and 10 °C. However, the addition of several molar equivalents of GAG octasaccharides greatly increased the number and intensity of detectable amine signals [57].

**Figure 13:**
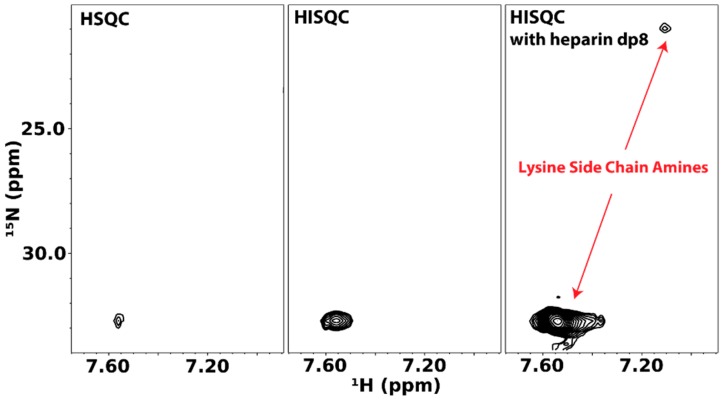
Lysine side chain amine ^15^N-HSQC and HISQC of pleiotrophin in the presence and absence of heparin octasaccharides. HISQC of the protein produced significantly stronger signals than HSQC. Addition of heparin octasaccharides also induced appearance of additional signals.

We have applied the same methodology to the cytokine pleiotrophin. Figure 13 shows lysine side chain amino HSQC and HISQC of pleiotrophin. The HISQC showed significant enhancements in intensity when compared to the HSQC. However, in both cases, signals can only be observed at pH 6.0 and low temperature (~10 °C). Unlike CXCL1/5, there is little dispersion in the lysine side chain signals of pleiotrophin, which reflect the fact that many of the lysines in pleiotrophin are located in the unstructured C-terminal tail. Similar to CXCL1/5, the presence of GAG dramatically enhanced the intensities of the lysine signals of pleiotrophin, indicating that interactions between GAG and protein likely reduced solvent exchange rates of amine protons. Despite the change in signal intensity, there is no significant change in the chemical shifts of the signals, which is indicative of highly dynamic interactions. 

Because GAG-induced chemical shift changes in lysine side chain signals can be small in many instances, it can be difficult to identify lysines that are involved in GAG binding by direct examination of the HISQC spectrum. Uhrin and co-workers showed that a more sensitive way to identify GAG-binding lysines is through direct cross-linking with GAGs [58]. To do this, they prepared GAG ligands whose carboxyl groups are activated with sulfo-*N*-hydroxysuccinimide. Activation of carboxyl groups on these ligands led to amidation reactions between the activated carboxyls on GAGs and nearby lysine side chains on proteins. The conversion of amine to amide eliminated signals of these lysines from the HISQC spectrum, allowing for them to be identified easily. It should be noted that the identity of these modified lysines can also be obtained through mass spectrometry. 

### 4.2. Specific Labeling of Lysine and Arginine Side Chains

Detecting intermolecular NOE cross-peaks between protein and GAG is usually quite difficult. Many factors contribute to this, including a lack of non-labile protons at the interface and the often dynamic nature of interactions between protein and GAGs. Compounding the difficulty is that the traditional ^13^C/^15^N-filtered/edited NOESY experiment used to obtain intermolecular contacts is low in sensitivity as a result of multiple isotopic filters and the need for ^13^C-labeling. To increase the sensitivity of the NOESY experiments, we have been exploring the use of deuterated protein containing specifically protonated lysines and arginines as a way to improve the sensitivity of the NOESY experiment to intermolecular contacts. By avoiding ^13^C-labeling, we increase the transverse relaxation time of protons and improve resolution in the both direct and indirect dimensions. However, a lack of ^13^C-labeling means spectral crowding cannot be resolved with ^13^C-edited experiments. Fortunately, specifically protonating the amino acids of interest while deuterating other amino acids mostly alleviate the problem. This method of specific labeling has the additional benefit of reducing spin diffusion and longitudinal relaxation, which can further enhance the sensitivity of NOESY to crucial long range intermolecular NOEs [59]. It also reduces transverse relaxation and eliminates some ^1^H homonuclear coupling, leading to additional increases in the signal-to-noise. However, it should be noted that the reduction in longitudinal relaxation requires increases in recycle delay time in order to re-establish the equilibrium magnetization.

**Figure 14:**
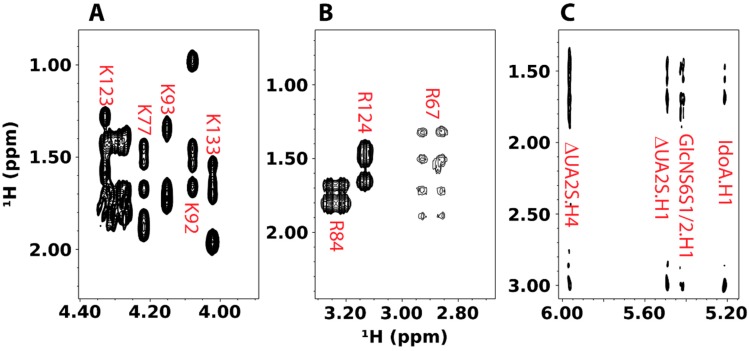
2D homonuclear NOESY spectra of deuterated pleiotrophin containing either ^1^H-labeled lysine or arginine. (**A**) Hα region of the NOESY spectrum of ^2^H-pleiotrohpin with specific ^1^H lysine labeling. Signals from lysines in the structured regions of pleiorophin are labeled in red. (**B**) Hδ region of the NOESY spectrum of ^2^H-pleiotrohpin with specific ^1^H arginine labeling. Signals from assigned arginines are labeled in red. (**C**) NOESY cross-peaks arising from intermolecular contacts involving GAG anomericprotons of 0.2 mM ^2^H-pleiotrophin with ^1^H lysine labeling in the presence of 2 mM heparin tetrasaccharide, ΔUA2S-GlcNS6S-IdoA2S-GlcNS6S.

To examine the efficiency and specificity of labeling, we have prepared ^2^H pleiotrophin containing either protonated lysines or arginines. Figure 14 shows the NOESY spectra of these samples. All lysines in the structured region of the protein can be identified. Most lysines have strong Hα signals, indicating that the deamination of the amino acid is not extensive. Attempts at specific labeling of arginine also produced relatively clean spectra, but Hα correlations of arginines are generally weak, a sign that the amino acid underwentsignificant deamination. Despite the weak Hα signals, correlations between other protons in the side chain are visible and three out of five arginines can be identified. Addition of heparin tetrasaccharide to the specifically lysine labeled sample showed that heparin can induce drastic changes in the Hα chemical shifts of K92 and K93, two lysines identified as crucial for GAG binding in mutagenesis studies. The higher resolution of homonuclear NOESY also allowed identification of K93 as having strong interactions with the uronic acids in the tetrasaccharide, but not glucosamine residues. In fact, both the non-reducing end unsaturated uronate and the iduronate close to the reducing end have similar contacts to K93. This indicates the tetrasaccharide is likely sliding along the GAG binding site and it does not have a single binding position. One remaining obstacle to the present methodology is that single lysine labeling is not possible. However, this can potentially be solved while using the amber codon technology and pyrollysyl-tRNAsynthetase, which allow for the incorporation of a single lysine terminated with the removable protecting group BOC.

## 5. Probing GAG-Induced Changes in Protein Dynamics and Oligomerization

**Figure 15:**
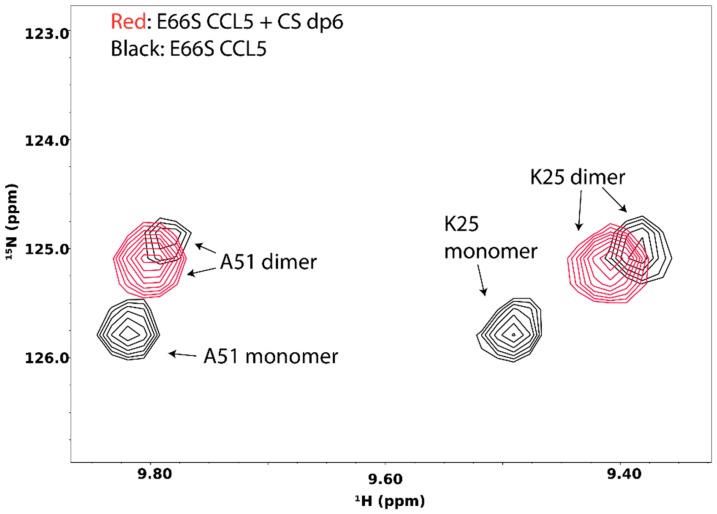
^15^N-HSQC spectra of CCL5 in the presence (red) and absence (black) of chondroitin sulfate hexasaccharide. At 40 °C, CCL5 exists in both dimer and monomer forms. However, addition of chondroitin sulfate hexasaccharide induces the formation of CCL5 dimer, leaving only dimeric CCL5 signals in the ^15^N-HSQC spectrum.

Interactions of GAG with target proteins often result in oligomerization and aggregation of the target. GAG-induced oligomerization is an important aspect of GAG’s activity and is crucial to the activation of many signaling proteins, including chemokines, growth factors, and membrane receptors. Knowledge of the protein oligomer formed in the presence of GAG is often vital to understanding the mechanism of signaling events. NMR is very sensitive to changes in protein oligomerization, which increases the transverse relaxation time and leads to peak broadening. In fact, by measuring spin longitudinal (R_1_) and transverse (R_2_) relaxation rates of protein complexes, the total correlation time of the complex can be calculated, and because MW is known to be correlated with the total correlation time, the size of the GAG-induced oligomer can be estimated. The detailed instruction on the procedure can be found on the website of the Northeast Structural Genomics Consortium (http://www.nmr2.buffalo.edu/nesg.wiki/NMR_determined_Rotational_correlation_time). In some instances, such as the case of the chemokine CCL5/RANTES, the monomeric and oligomeric species are in slow exchange on the NMR timescale under the right conditions, and therefore can be separately identified in the NMR spectrum (Figure 15). In this particular case, the disappearance of the monomeric species in the presence of chondroitin sulfate hexasaccharide offered conclusive evidence that GAG-induced oligomerization has occurred. 

Besides the use of global dynamics in the characterization of GAG-induced oligomerization, quantitative characterization of GAG-induced local changes in protein dynamics can also be done. NMR is an excellent tool for studying protein dynamics at a wide range of timescales. For fast motion on the ps-to-ns timescale in ^15^N atoms, the model-free formalism can be used to estimate local motion of individual atoms using their heteronuclear NOE, R_2_, and R_1_ relaxation rates [60]. For slow motion on the µs-to-ms timescale, relaxation dispersion experiments can be used to estimate the frequency of the motion as well as the change in chemical shift of the atom as a result of the motion. Such analyses have been carried out on several protein-GAG systems. One example comes from the study of vascular endothelial growth factor-165 (VEGF_165_) by Kim et al. [61]. They looked at the effects of GAGs on slow timescale motions of ^15^N atoms in the protein backbone in heparin binding sites of VEGF_165_ while using relaxation dispersion experiments. They noticed that many residues in the GAG-binding sites exhibit dynamic motion in the ms timescale, but these motions were decreased dramatically by the presence of heparin octasaccharide, indicating heparin has stabilized the GAG-binding site. 

On the other hand, Feng et al. looked at GAG-induced slow timescale motion of decorin-binding protein B (DBPB), a GAG-binding surface adhesin that is found on the Lyme disease bacterium *Borrelia burgdorferi*. They found that GAG binding does not change the ps-to-ns timescale motion of DBPB. However, dermatan sulfate oligosaccharides were able to induce slow timescale motion of the residues in the GAG-binding site [46]. Interestingly, this phenomenon is only seen with dermatan sulfate and not other GAGs, such as heparin, indicating that the kinetics of interaction between DBPB and GAG is GAG-type specific. Methods also exist to look at the relaxation of the ^15^N atom in lysine side chain amine groups. They have been productively applied to the study of DNA-binding proteins [55,56]. Although there is currently no report of application of these methods to GAG-binding proteins, the utilization of these methods on GAG-binding proteins should shed important insights into protein-GAG interactions.

## 6. Solid-State NMR Studies of GAG-Protein Interactions

One important biological activity of GAG is their ability to induce formation of amyloids. In fact, GAG is known to promote amyloid formation of a number of proteins, including Aβ40/42, α-synuclein, tau, amylin, and calcitonin [62,63,64,65,66]. Because the aggregates of these proteins are beyond the size regime of solution NMR and are not capable of forming crystals, solid state NMR has become a favored tool for investigatinginteractions of GAG with amyloids. One particular notable series of studies was carried out by the groups of David Middleton and Sheena Radford [67,68,69,70]. They discovered that low-MW heparin only associates strongly with Aβ40 amyloid, and not Aβ42 or shorter peptides. The structure of the amyloid was also found to be important as amyloid with three-fold cross-sectional symmetry has significantly higher affinity for heparin than those with two-fold symmetry. Using solid-state NMR and a synthetic ^13^C-enriched heparin octasaccharide, they determined that the octasaccharide remains rigid and is in contact with the amyloid throughout. They also examined amino acids involved in binding heparin by looking at signal dephasing caused by dipolar interactions between ^15^N atoms in lysine, arginine and histidines, and ^13^C atoms in the heparin octasaccharide. Surprisingly, the contact between heparin and the amyloid was mediated mostly by histidine residues. Since most of the histidines are located at the junction between peptides in the same cross-sectional layer, they were able to construct a model of these GAG-Aβ40 amyloid complexes. The results also explained the lower heparin affinity of Aβ40 amyloids with two-fold symmetry, since the arrangement of amino acids in two-fold symmetric structures did not allow for correct clustering of the basic amino acids. It should be noted that the availability of ^13^C-labeled heparin ligands was crucial to their studies. Without this ligand, the important frequency selective rotational echo double resonance experiment used to measure dipole-dipole interactions between ^15^N and ^13^C atoms would not be possible.

## 7. Computational Modeling of Protein-GAG Complexes Using Sparse Constraints

Obtaining a large quantity of experimental high-resolution structural information on GAG-protein systems is usually not feasible. As a result, constructing of models of protein-GAG complexes cannot be accomplished while using conventional NMR structure determination protocols. This is because the default simulation conditions in these procedures use simplified force-fields in either implicit solvent or vacuum models. Although these parameters greatly expedite the calculation, they require more experimental information to arrive at the correct structure, which is unattainable for most protein-GAG systems. The available force field parameters for creating GAG ligands are also lacking in these software. For instance, molecular topology specification in XPLOR-NIH [71] and CNS [72] cannot easily be used to define the puckering of the sugar ring. However, IdoA residues in most GAG ligands adopt the ^1^*C*_4_ rather than the traditional ^4^*C*_1_ puckering. The inability to consistently specify ring puckers means that the generation of the correct ligand structure using the software is usually cumbersome. The imprecision in the carbohydrate parameters also means longer calculation times are required to find the minimal energy conformation of the ligand in some cases. This makes obtaining ligand structures that are consistent with experimental data difficult. On the other hand, although fully fledged molecular dynamics simulation software usually have more accurate force field and realistic simulation conditions, the relatively large size of most protein-GAG systems means that unrestricted molecular dynamics simulation is not likely to produce accurate structures in a realistic time frame. 

**Figure 16:**
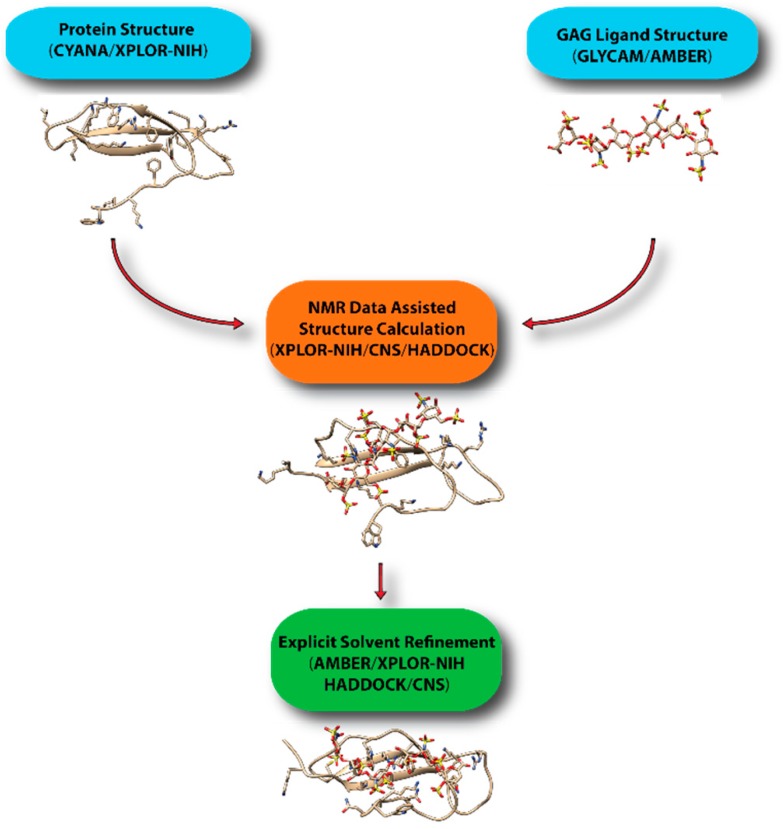
Common workflow in determining structures of protein-GAG complexes using sparse information. To ensure proper ring puckering and stereo chemistry, the initial ligand structure should be created with AMBER using the updated GLYCAM force field. After docking the ligand using experimental data in either XPLOR-NIH or HADDOCK/CNS, an explicit solvent refinement is highly desirable.

To overcome these limitations, we have been using a hybrid approach to create realistic models of protein-GAG complexes. In particular, we first obtain the coordinate of the GAG ligands while using AMBER and its GLYCAM force field, which has just been updated to include monosaccharides specific to GAGs [73]. This step also allows us to put the ligand in the correct bound-conformation prior to structure calculation. We then use the structure in XPLOR-NIH or CNS to dock the glycan onto protein using NMR constraints. The lowest energy structures are then further refined in AMBER. Other groups have adopted similar protocols in which an approximate model is constructed while using traditional NMR software, such as XPLOR-NIH, CNS, and HADDOCK [74], and further refined under more realistic environments in molecular dynamics simulations if necessary. Figure 16 displays a general scheme for the workflow in computational modeling of GAG-protein complexes while using NMR data. GAG-binding proteins studied using such an approach include CXCL1 [75], CXCL5 [76], β-defensin [21], Robo-1 [77], and LAR [31]. HADDOCK has proven to be especially popular because of its ability to incorporate a large array of ambiguous data from NMR, including information from chemical shift perturbation, ambiguous NOEs, residual dipolar couplings and pseudo-contact shifts. It should be noted that there are several docking softwarethat are designed specifically for protein-GAG systems [78,79,80,81]. However, it is not clear whether they can easily incorporate experimental constraints from NMR.

## 8. Conclusions

GAG-dependent regulation of proteins is a pivotal biochemical process in many physiological and pathological events. The understanding of the resultant GAG-protein complexes at the atomic level is necessary to better comprehend the underlying mechanisms that are involved in these events and makes the design of new drugs targeting these proteins possible. NMR spectroscopy is a versatile and robust analytical technique that is often employed in the studies of GAG-protein interactions. Through isotopic labeling, not only is NMR capable of examining the physicochemical properties of the complexes as a whole, but it also excels at examining changes affected by its binding partner in each individual component. Multiple NMR methods are available or under development for assessing the characteristics of the GAG-protein complexes. Here, we reviewed the theoretical and practical aspects of the most common methods that are found in the literature. The experiments discussed herein were CSP, STD, trNOE, PRE, and waterLOGSY. In addition, more recent methods utilized to assess contribution of arginine and lysine side chains in interactions with GAGs and computational approaches commonly employed to model GAG-protein interactions while using NMR-derived information are also presented. We also discussed the importance of measuring spin relaxation rates for assessing dynamic information of the GAG-protein complexes in solution as well as the preparation of GAG oligosaccharides of defined chemical structures for isotopic and paramagenetic labeling so they can be used as functional probes in GAG-protein interaction studies. The number of investigations in this field has increased considerably during the last few years. These methods have been proven to be essential not only to the structural biologists, but also offer benefits to those professionals working in drug discovery. In this regard, we believe that NMR spectroscopy will continue to play a key role in future investigations of GAG-protein interactions.

## Figures and Tables

**Table 1:** Commonest monosaccharides and sulfation patterns of the different (sub)classes of glycosaminoglycans (GAGs).

GAG Type or Sub-Type	Hexosamine *	Uronic Acid or Neutral Sugar *	Sulfation Pattern *
Heparin	4-linked α-GlcNAc	4-linked α-IdoA	GlcNS6S, IdoA2S
Heparan sulfate	4-linked α-GlcNAc	4-linked β-GlcA	non-sulfated
Chondroitin sulfate A	3-linked β-GalNAc	4-linked β-GlcA	GalNAc4S
Chondroitin sulfate B (dermatan sulfate)	3-linked β-GalNAc	4-linked α-IdoA	GalNAc4S, IdoA2S
Chondroitin sulfate C	3-linked β-GalNAc	4-linked β-GlcA	GalNAc6S
Chondroitin sulfate D	3-linked β-GalNAc	4-linked β-GlcA	GalNAc6S, GlcA2S
Chondroitin sulfate E	3-linked β-GalNAc	4-linked β-GlcA	GalNAc4S6S
Keratan sulfate	4-linked β-GlcNAc	3-linked α-Gal	GlcNAc6S, Gal6S
Hyaluronan	3-linked β-GlcNAc	3-linked β-GlcA	non-sulfated

* The abbreviation of the sugars are GlcNAc, *N*-acetylglucosamine; IdoA, iduronic acid; GlcNS6S, *N*,6-di-sulfated glucosamine; IdoA2S, 2-sulfated iduronic acid; GalNAc, *N*-acetylgalactosamine, GalNAc4S, 4-sulfated *N*-acetylgalatosamine; GalNAc6S, 6-sulfated *N*-acetylgalactosamine; GlcA2S, 2-sulfated glucuronic acid; GalNAc4S6S, 4,6-di-sulfated galactosamine; Gal, galactose; GlcNAc6S, 6-sulfated *N*-acetylglucosamine.

**Table 2:** Nuclear Overhauser effect (NOE)- and trNOE-derived interproton distances of Fx free and bound to LAR-Ig1-2. Adapted with permission from Gao et al. Biochemistry 2018, 57, 2189–2199. Copyright. 2018 American Chemical Society [31].

^1^H-^1^H Connection ^a^	Free ^b^	Bound
AH1-BH4	2.62 ± 0.02 ^c^	2.86 ± 0.21
CH1-DH4	2.41 ± 0.02	2.65 ± 0.20
CH3-DH1	2.43 ± 0.11	3.05 ± 0.25
DH1-DH2	3.16 ± 0.01	-
DH1-DH3	3.27 ± 0.01	2.72 ± 0.19
DH1-EH4	2.71 ± 0.02	3.14 ± 0.25
DH1-EH6	2.85 ± 0.02	2.91 ± 0.23
DH1-EH6′	3.22 ± 0.03	2.49 ± 0.19
*O*Me-EH1	2.96 ± 0.02	3.22 ± 0.20

^a^ The structure and nomenclature used were GlcNS6S-GlcA-GlcNS3S6S-IdoA2S-GlcNS6S-*O*Me and A-B-C-D-E-*O*Me. ^b^ The distance of 2.50 Å between H_2_ and H_4_ of the GlcNS6S ring A was used reference to calculate the other NOE- and trNOE-derived interatomic distances. ^c^ Errors were estimated based on the signal-to-noise of the individual peaks.
